# Simplified flow cytometric quantification of human neutrophil extracellular traps (NETs)

**DOI:** 10.1186/s12950-026-00490-0

**Published:** 2026-02-18

**Authors:** G. Rinaldi, K. K. W. Cheng, C. McCann, F. Rossi, M. Rodriguez-Rios, K. Dhaliwal, A. Bebes, L. Duncan, R. Yuecel, Adriano G. Rossi, Calum T. Robb

**Affiliations:** 1 Centre for Inflammation Research, Institute for Regeneration and Repair, University of Edinburgh, Edinburgh, Scotland, EH16 4UU UK; 2 Centre for Regenerative Medicine, University of Edinburgh, Edinburgh, Scotland, EH16 4UU UK; 3 EaStCHEM School of Chemistry, Joseph Black Building, University of Edinburgh, Edinburgh, Scotland, EH9 3FJ UK; 4 Iain Fraser Cytometry Centre, Institute of Medical Sciences, University of Aberdeen, Aberdeen, Scotland, AB25 2ZD UK; 5 Present Address: Department of Physiology, Development and Neuroscience, University of Cambridge, Downing Site, Cambridge, CB2 3DY UK; 6 Present Address: Centre for Cytomics, Centre for Life Science Technologies, Henry Wellcome Building for Biocatalysis, University of Exeter, Exeter, EX4 4QD UK

**Keywords:** Neutrophil extracellular traps, Flow cytometry, Imaging flow cytometry, NET, NET detection, NET quantification, NETosis, NADPH oxidase, Calcium ionophore

## Abstract

Several cellular pathways lead to the formation of neutrophil extracellular traps (NETs), a form of cell death (NETosis), distinct from apoptotic and necrotic cell death. Surprisingly, there remains a paucity of methods enabling efficient quantification of NETosis and associated pathways. Here, we describe the development of a simple, sensitive, reliable and flexible flow cytometry assay allowing efficient detection and quantification of NETosis. For the core assay, isolated primary human neutrophils were incubated with stimulants e.g. PMA/ionomycin with or without inhibitors prior to fixing. The fixed cells were then blocked and subsequently incubated in anti-DNA/Histone 1 and anti-histone H2A antibodies for dual detection of Histone 1 and Histone H2A. Cells with were H1-DNA/H2A double fluorescent were deemed NETotic. Imaging flow cytometry was used to validate the accuracy of NETosis detection/quantification. Several key pathways of NETosis were confirmed via the use of established NET-inducers in accordance with existing established methodologies. Importantly, our novel flow cytometry-based NETosis detection assay could efficiently discriminate NETosis from established neutrophil activation, as well as apoptotic and necrotic cell death. We believe our methodology will complement existing NETosis methodologies whilst concomitantly reducing human error, subjectivity and, indeed the false positivity (attained from neutrophil activation and other cell death processes) inherent in existing methodologies. Furthermore, the simplicity and flexibility of our methodology permit additional markers and pathways of NETosis to be investigated, highlighting it as an integral research tool for both general NETosis research and the pursuit to better understand the pathogenesis of NETosis-associated diseases.

**Supplementary Information:**

The online version contains supplementary material available at 10.1186/s12950-026-00490-0.

## Graphical Abstract

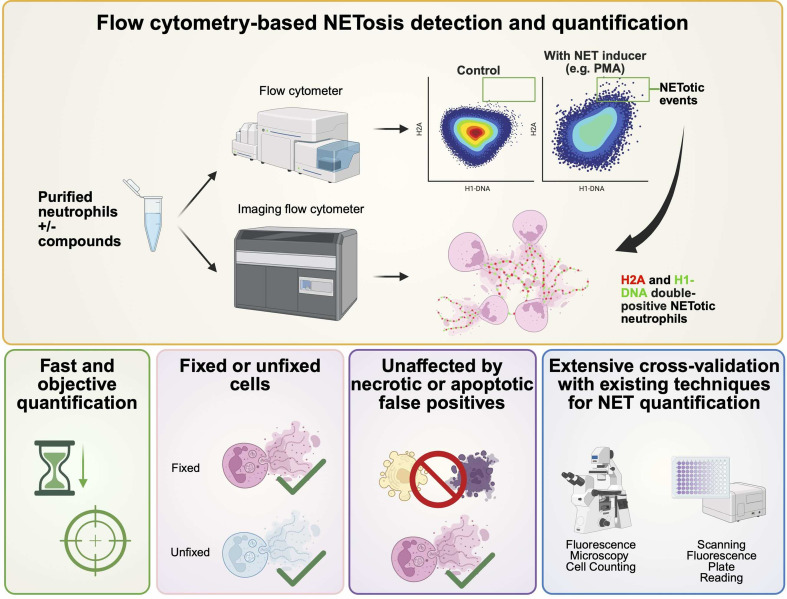

## Background

Neutrophil extracellular traps (NETs) [1] occur collectively during NETosis [2], a form of cell death distinct from apoptotic and necrotic cell death [3]. Lytic NETosis involves the controlled extrusion of decondensed nuclear chromatin into the immediate surrounding extracellular space, studded with nuclear, granule and cytosolic proteins, which ensnare and kill invading microorganisms [1, 3–5]. Furthermore, NETosis confers highly localised antimicrobial activity [6], initiates and regulates coagulation cascades [7], as well as play integral roles during anti-inflammatory activities, including immunologically silent clearance of NETs by macrophages [8] and limiting inflammation (during gout) via cytokine and chemokine degradation [9]. Conversely, aberrant NETosis can contribute to a wide range of NETosis-associated diseases. These include various autoimmune diseases such as systemic lupus erythematosus (SLE) [10] and rheumatoid arthritis [11], as well as sepsis [12–16] and cystic fibrosis [17]. NETosis has also been implicated in promoting cancer metastasis to healthy tissues [18, 19] and awakening dormant cancer cells [20]. Interestingly, NETosis is not confined to neutrophils, leading to the collective term ETosis [21] and is indeed an evolutionary ancient and conserved cell death process found in immune cells of lower invertebrates [22], serving to explain some of its adverse effects observed in mammals. Given the multiplicity of NETosis-associated diseases that exist, it is therefore of vital importance that NETosis detection and quantification methods are sensitive, reliable and above all, accurate.

Generally, NETs are most commonly examined in monolayers following in vitro culture using cell culture vessels. The gold standard NET detection methods include scanning electron microscopy to identify smooth fibres and globular domains/complexes on three-dimensional extracellular DNA networks [1], and transmission electron microscopy to detect hallmark nuclear membrane fragmentation and blending of nuclear, cytoplasmic and granular constituents [3]. Immunocytochemistry and immunohistochemistry are also commonly deployed for detecting key NETosis markers such as histones and neutrophil elastase [1, 3, 23]. However, such methods are commonly considered qualitative and not quantitative of NETosis.

At present, there are several methodologies used extensively for the quantification of NETosis, including fluorescence microscopy counts, scanning fluorescence plate readings and enzyme-linked immunosorbent assays (ELISAs). While these approaches are widely applied, each has notable limitations. Microscopy-based methods are inherently subjective and require expertise to distinguish NET structures [24]. To circumvent this, computational algorithms have since been developed and used to automate the quantification of NETosis using fluorescence microscopy and, therefore, reduce human error and bias [24, 25]. Scanning fluorescence plate detection of NETotic DNA via the use of a single vital DNA stain (e.g. Sytox Green) [26] can be susceptible to false positives from other forms of cell death e.g. late apoptosis/necrosis. ELISAs detecting NET-specific myeloperoxidase (MPO)-DNA, human neutrophil elastase (hNE)-DNA products [27, 28] or citrullinated histone H3 [29], can be performed using many commercially available ELISA kits but they do not capture the structural complexity of NETs.

Flow-cytometry based approaches have been reported [30–35] but these reports use broadly varying methodologies in terms of sample preparation and gating strategies used for NETosis detection/quantification. Additionally, many of these reports did not investigate the mechanisms underlying the quantified NETotic events, nor did they ensure that false positives from activated, necrotic, or apoptotic cells were minimized [36]. In cases where such mechanisms were examined, the approaches used were not thoroughly explored or validated with established methods. Hence, there is demand for a simple and standardised flow cytometric approach to quantify qualitative NETotic events and associated mechanisms, that will help complement established methodologies. Flow-cytometric based assays are advantageous over imaging-based assays due to its inherent objective nature.

While flow-cytometry based NETosis detection has been described, varying methodologies of sample preparation and gating strategies for NETosis detection and quantification were employed in these reports [30–32, 37]. Here we report the design, validation and accurate detection/quantification of lytic NETosis using a simple flow cytometric approach. Our methodology is centered on dual or triple detection of key NETosis markers through antibody staining - such as DNA/Histone H1 and Histone H2A - or with smartprobes like HNE-FQ (detection of human neutrophil elastase, hNE) [38], which enhance the specificity of NETotic event identification. The linker histone, H1, has been shown to be present on phorbol 12-myristate 13-acetate (PMA) - and A23187 (a calcium ionophore)- induced NETs [39–41], and the anti-DNA/Histone H1 antibody exhibits strong affinity for decondensed chromatin on NETs [42]. Antibodies against H2A were also incorporated in our assay as it makes up the highest proportion (26.9%) of NET proteins [43].

We validated the sensitivity and specificity of our assay by studying widely used non-physiological NET inducers (PMA and the calcium ionophore ionomycin) and biologically relevant inducers (*Staphylococcus aureus)* and studied their specificity using various specific NET inhibitors. PMA-induced NETosis, a well-established inducer of lytic NETosis [3, 44], is dependent upon activation of PKC [45] and nicotinamide adenine dinucleotide phosphate (NADPH) oxidase [3]. Such PMA-induced NETosis can be inhibited via utilisation of diphenyliodonium (DPI) [3], which inhibits flavoprotein oxidoreductases (including NADPH oxidase) and Ro 31-8220, a pan-PKC inhibitor which inhibits generation of reactive oxygen species (ROS) upstream of NADPH oxidase activation [45]. To investigate non-NADPH dependent NETosis, neutrophils were treated with ionomycin, that induces NET formation through activation of voltage-independent Ca^2+^ – activated K^+^ (SK) channels via the generation of mitochondrial ROS [46]. Ionomycin-induced NETosis can be inhibited by pan-selective SK channel inhibitor, apamin, and the mitochondrial uncoupler, 2,4-Dinitrophenol (DNP) [46].

Our assay was also validated on both live or fixed samples and samples in suspension or adhered to plates which will vastly improve the flexibility of using flow-cytometry to study NETosis. To ensure consistency in findings, we compared results obtained using conventional scanning fluorescence and fluorescence microscopy plate-based assays with those obtained using our flow-cytometry based assay.

## Methods

### Ethics statement and human neutrophil isolation

Human neutrophils were isolated from healthy blood obtained from healthy volunteers under the ethical approval obtained from the Lothian Research Ethics Committee #08/S1103/38 as previously described [47]. Briefly, 40mL peripheral blood was drawn and mixed with 4 mL of 3.8% sterile sodium citrate tribasic dihydrate. After centrifugation at 350 g for 20 min at acceleration and breaking gauges set to 0, the platelet-rich plasma (PRP) was removed, 10 ml of which was added to 220 µL of 1 M calcium chloride and left for 1 h at 37 °C in a water bath to form a platelet plug and autologous serum. The leukocytes were isolated from the remaining cells using dextran sedimentation. After washing the leukocytes in PBS without Ca^2+^ and Mg^2+^ (-/-) by centrifugation at 350 g for 6 min, the leukocyte pellet is then resuspended in 55% Percoll solution and the neutrophils are isolated using a discontinuous Percoll gradient. Neutrophil purity was assessed using cytocentrifugation and only neutrophil isolations of extremely high purity were utilised for further experiments.

### In vitro culture of neutrophils

Isolated neutrophils were resuspended in RPMI 1640 (Gibco) containing 5% FBS at 1 × 10^6^ mL^−1^. Neutrophils were incubated with various combinations of NET stimulators including 10 nM PMA (Sigma) or 5 µM ionomycin (Sigma) for 3 h with or without pre-treatment using inhibitors including 10 µM DPI (Sigma), 1 µM Ro 31-8220 (Sigma), 750 µM DNP (Sigma), 100 µM Sivelestat and 200 nM apamin (Tocris) for 30 min - all established in vitro culturing times for inducing NETs with PMA and ionomycin. To investigate the effect of activated neutrophils on assay reliability, neutrophils were incubated with 100nM fMLF or 1 µM A23187 for 30 min at 37 °C. To stain nucleic acids, 1 µM of Sytox Green (Invitrogen) was added and incubated with the cells for 20 min.

### Bacterial culture

*Staphylococcus aureus* (ATCC-Seattle 1945), a gram-positive bacterium, was utilised to stimulate NET formation [48, 49]. *S. aureus* was cultured in Luria-Bertani (LB) broth overnight at 37 °C in a shaking incubator. 100 µL of the overnight culture was added to 10 mL of fresh LB broth and incubated at 37 °C until the OD at 600 nm reached 0.6. A multiplicity of infection of 20 (MOI = 20) was cultured with isolated neutrophils − 500,000 neutrophils with 10 million *S. aureus* bacteria.

### Fluorescent microscopy counts

The cells were imaged using the EVOS FL Auto2 fluorescent microscope (Thermo Fisher Scientific) at 40X. The images were analysed using the cell counter plug-in in ImageJ (National Institutes of Health) where a minimum number of 300 cells were counted from a minimum of 3 random fields while avoiding the well edges. NETs which were identified by their hallmark morphological features were quantified as a percentage of the total number of cells present for each treatment.

### Scanning fluorescent plate reading

To interrogate real-time NET development, cells were incubated with Sytox Green 1 µM at the start of the experiment and fluorescence measured using a Synergy H1 Hybrid microplate reader (BioTek) using the Gen5 software. The program was set to ‘scanning’ which calculates the average fluorescence of 10 set areas from each well. Images were taken at 30-min intervals up to 4 h.

### Flow cytometry

For fixed samples, after treating the neutrophils with stimulators/inhibitors, they were fixed in an equal volume of 1% paraformaldehyde for 30 min at 37 °C and then centrifuged at 15,000 g for 20 min. After removal of the supernatant, the cell pellet is resuspended in 2% BSA and left to block overnight at 4 °C. Following this, the cell pellets were washed and resuspended in 2% BSA supplemented with mouse anti-DNA/Histone 1 monoclonal antibody (H1-DNA; 1:2,500; Millipore) for 1.5 h at room temperature followed by GAM Alexa-488 (1:2,500; Abcam) to label H1-DNA and an anti- histone H2A-Alexa-647 conjugated antibody (H2A; 1:2,500; Abcam) to detect histone H2A for a further 1.5 h. For live samples, cells were stained with 200nM Sytox Green and anti-histone H2A-Alexa-647 conjugated antibody (1:2,500) for 30 min at 37 °C. Prior to flow cytometry analysis, cells were centrifuged as before and resuspended in 150 µL of PBS (-/-). The Novocyte flow cytometer (ACEA Biosciences Inc) were used to run and analyse the samples. For live cells, only cells exhibiting high double fluorescence for Sytox Green and H2A-Alexa-647 were gated and considered for data analysis.

To study the effect of apoptosis and necrosis on our assay reliability, neutrophils were incubated with 10 µM of the pan-caspase inhibitor, Q-VD-OPh (QVD; rndsystems) or 50% ethanol for 6 and 24 h at 37 °C. At each time point, cells were resuspended before 30 µL cells were added to 200 µL Annexin V binding buffer (HBSS containing Magnesium and Calcium supplemented with 5 mM CaCl_2_) containing Annexin V-FITC (AV; 1:500; Roche) and left on ice in the dark for 10 min. 1 µL of 1 mg mL^-1^ Propidium iodide (PI; Sigma) was added just prior to running the samples on the FACSCalibur (BD Biosciences) which was programmed to detect viable (both AV and PI negative), early apoptotic (AV positive and PI negative) and late apoptotic/necrotic cells (Positive PI with positive or negative AV). With each experiment, samples were also analysed using our flow cytometry assay. For flow cytometry analysis of NETs at both 6 and 24 h, gates to measure high fluorescing events double positive for both H1- DNA/H2A were drawn relative to QVD treated samples as it was confirmed from the AV/PI assay that > 95% neutrophils remained viable after these time points.

### Imaging flow cytometry

To confirm that the cells identified as high fluorescing events double positive for key NET makers using our novel flow cytometry assay were indeed NETotic, fixed cells were analysed using a ImageStreamX mark II imaging flow cytometer (Amnis, Merck Millipore). NETotic cells were identified by their classically described features of large nuclear area/staining H1-DNA/H2A, overlapping regions of H1-DNA/H2A and the degree of colocalization of H1-DNA/H2A.

### Cytotoxicity assay

Lactate dehydrogenase (LDH) release was using a cytotoxicity detection kit (Roche) as a marker of cytotoxicity. Briefly, as a positive control, cells were treated with 4% Triton X (Thermo Fisher Scientific), and as a negative control, supernatant from untreated cells or media alone was used. The supernatant from cells cultured with or without HNE-FQ was collected. Just prior to use, the reaction mixture from the kit was prepared according to manufacturer’s instructions. After adding the reaction mixture to the samples and incubating for 30 min in the dark at room temperature, the absorbance of each sample was read with a Cytation 3 plate reader (Bio Tek) at a wavelength of 490 nm. The following formula was used to calculate the percentage of cytotoxicity.

$$\begin{aligned}&Cytotoxicity\:\left(\%\right)\cr & \quad=\frac{experimental\:value-negative\:control\:value}{positive\:control\:value-negative\:control\:value}\cr & \quad\times\:100\end{aligned}$$

### Quantification and statistical analysis

Data were calculated as the mean of at least three different donors in three (*n* = 3) experiments. Statistical differences were determined using either one-way or two-way ANOVA or two-tailed paired T-test using Prism (GraphPad) with significance defined as a P-value < 0.05. A Student-Newman-Keuls (for one-way ANOVA) or Bonferroni (for two-way ANOVA) test was utilised for post-hoc multiple comparisons where ANOVA revealed a *P* < 0.05.

## Results

### Development of a flow-cytometry-based NETosis detection assay and validation using imaging of identified NETs

**Fig. 1:**
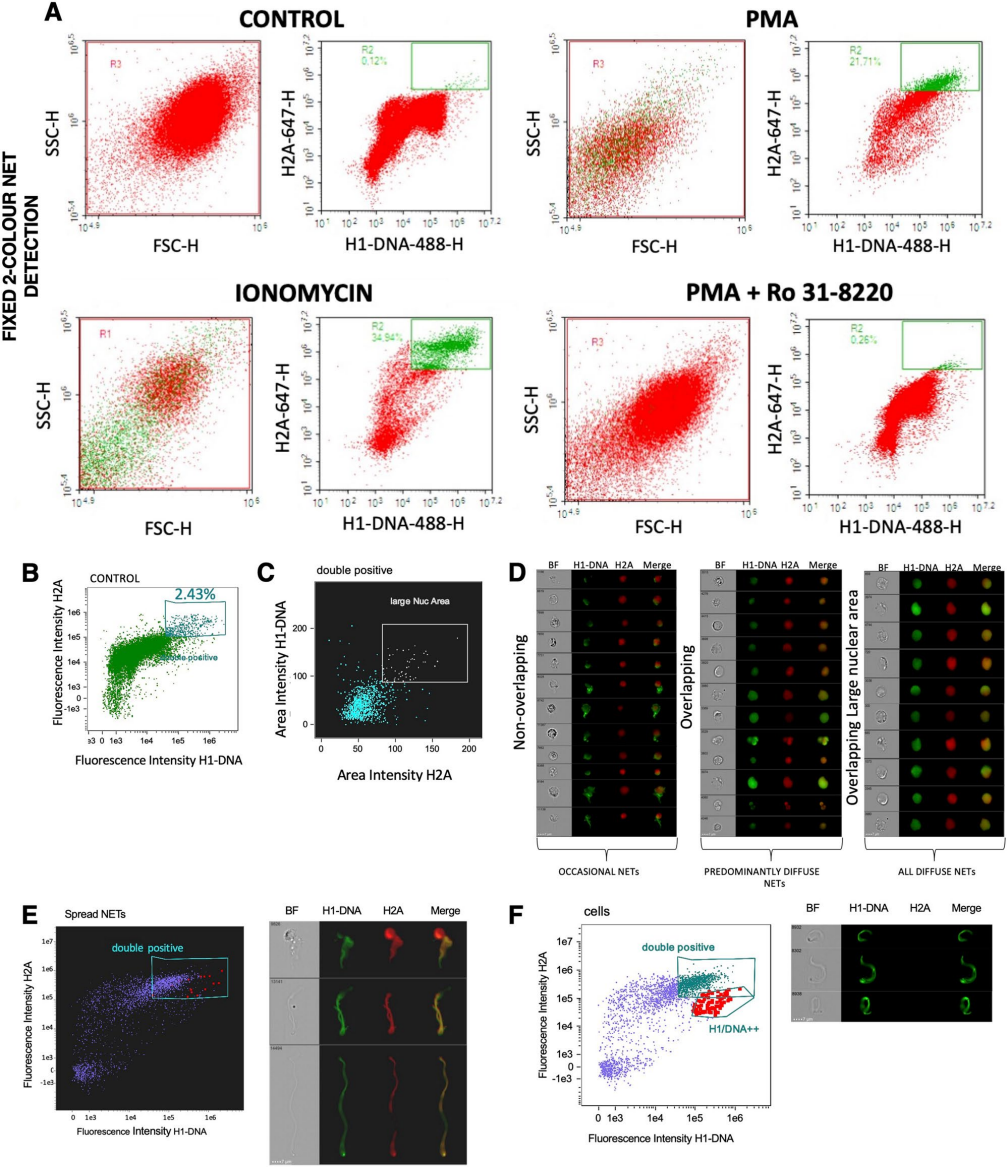
Characterisation and validation of the flow-cytometry-based NETosis assay with imaging flow cytometry. (**A**) Representative flow-plots after a 3 h incubation without or with 10 nM PMA and 5 µM ionomycin +/- 10 µM DPI and 1 µM Ro 31-8220 (PMA- specific NET inhibitors). *n* = 3. (**B**) NETotic cells identified as H1-DNA and H2A double-positive population. (**C**) Cells with large nuclear area defined as H1-DNA and H2A with high area intensity. (**D**) Representative images of PMA treated cells in the non-overlapping, overlapping and overlapping large nuclear area populations. Scale = 7 μm. (**E**) Flow cytometric identification and representative images of double positive (H1-DNA and H2A) spread NETs, with individually identified NETotic cells highlighted in red. Scale = 7 μm. (**F**) Flow cytometric identification and representative images of H1-DNA only diffuse NETs, with individually identified NETotic cells highlighted in red. Scale = 7 μm

For our flow-cytometry-based NETosis detection assay, firstly, a generous gate was drawn over the entire dot plot of forward scatter (FSC) vs. side scatter (SSC) of viable control (untreated) neutrophils (Fig. 1A) per donor. Doublets were not excluded due to the “sticky” properties of NETs. Cells were stratified according to the H1-DNA/H2A fluorescence intensity (Fig. 1B) and area intensity (Fig. 1C).

Control neutrophils were further analysed on dot plots of H1-DNA-488-H vs. H2A-647-H fluorescence. A rectangular gate was drawn which commenced approximately at 10^4^ H1-DNA-488-H and encompassed the remaining top right corner region of the dot plot, free of cellular events. This gate, ascertained from the control sample, was then applied to all treatments for that specific donor and the mean percentage NETotic events and mean fluorescence intensities of H1-DNA-488 and H2A-647. The gate was set for each experimental round based on the control sample, and any events falling within this region were classified as high-fluorescence H1-DNA and H2A^++^ and thus considered NETotic (Fig. 1A). Imaging flow cytometry confirmed that the majority of H1-DNA and H2A^++^ events deemed NETotic by our conventional methodology were in fact ‘true’ NETotic events (Fig. 1D). Moreover, most diffuse NETs and spread NET fragments remained sufficiently intact during sample preparation (Fig. 1E and F) which involved several high-speed centrifugations. Furthermore, these NETs appear very similar in morphology to NETs previously analysed by imaging flow cytometry [33–35]. After successful confirmation of our methodology using imaging flow cytometry, we now had a high degree of confidence that our flow cytometric assay for NETosis detection/quantification was indeed sensitive, accurate and reliable.

### Flow-cytometric based NETosis detection assay validated using established NET inducers, PMA and ionomycin

To validate our flow-cytometric based NETosis detection assay, we opted to induce NET production using PMA and ionomycin.

**Fig. 2:**
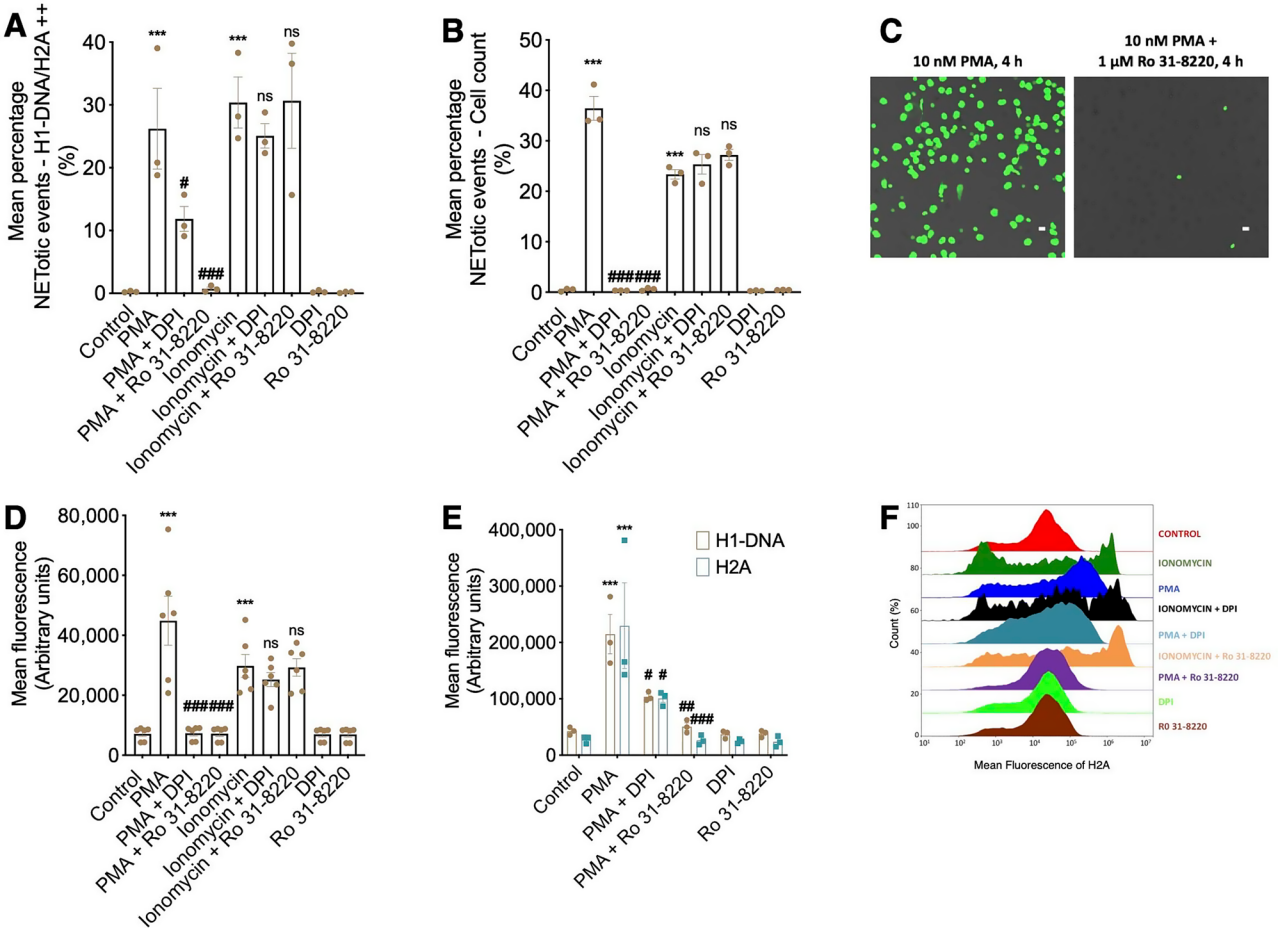
Flow cytometric detection and quantification of established PMA-induced NETosis dependent upon activation of conventional PKC and NADPH oxidase. (**A**) Mean percentage NETotic events defined by H1-DNA/H2A double fluorescence after a 3 h incubation without or with 10 nM PMA and 5 µM ionomycin +/- 10 µM DPI and 1 µM Ro 31-8220 (PMA- specific NET inhibitors). *n* = 3 distinct cell donors. (**B**) Mean percentage NETotic events quantified using cell counting after 4 h incubation. *n* = 5. (**C**) Representative images of neutrophils treated with PMA or PMA and Ro 31-8220 after 4 h of incubation, stained with Sytox Green. Scale = 10 μm. (**D**) Mean fluorescence of Sytox green-stained neutrophils quantified using scanning fluorescence after 4 h incubation with various mediators. *n* = 3 distinct cell donors. (**E**) MFI quantification of H1-DNA and H2A after a 3 h incubation without or with 10 nM PMA and 5 µM ionomycin +/- 10 µM DPI and 1 µM Ro 31-8220 (PMA- specific NET inhibitors). *n* = 3 distinct cell donors. (**F**) Representative stagger offset histogram of H2A fluorescence with various incubations. Data are expressed as means +/- SEM. **P < 0.01, ***P < 0.001 vs. corresponding control; # *P* < 0.05, ## *P* < 0.01, ### *P* < 0.001 vs. corresponding PMA; ns vs. ionomycin

As expected, PMA- and ionomycin-treated cells had significantly increased double-positive H1-DNA/H2A population (NETotic events) compared to control (Fig. 2A). To further validate our assay findings, we also quantified NETosis in these experiments using fluorescent microscopy counting (Fig. 2B) and scanning fluorescence plate reading (Fig. 2C and D), both well-established quantification methods, demonstrating similar patterns to ones observed using our flow-cytometry assay. In contrast, when PMA-treated cells were pre-incubated with specific inhibitors, DPI (NADPH inhibitor) or Ro 31-8220 (PKC inhibitor), a reduction in this population was observed. Consistent with the described literature, this population was not decreased in ionomycin-treated cells, which were pre-treated with these inhibitors, demonstrating conservation of pathway specificity. The mean fluorescent intensity of H1-DNA and H2A demonstrated a similar pattern of significantly increased intensity in PMA-treated cells which were reduced in PMA-inhibitor pre-treated cells (Fig. 2E). A clear shift in H2A fluorescence intensity is observed in an overlay of H2A histograms (Fig. 2F).

While these experiments have been performed on fixed non-adhered cells, we also demonstrate that the assay is sensitive at identifying NETotic events in adhered cells post-monolayer culture (Supplementary Fig. 1A and B) and unfixed cells (Supplementary Fig. 1C and D).

Other hallmark NET-proteins utilised include hNE which is an essential requirement for NETosis [50], and MPO, which blocks NET production when absent [51]. As such, when either hNE or MPO were added to our existing NET-marker panel, we adjusted the fluorochromes accordingly to ensure minimal spectral overlap was observed during tricolour NET detection. NET identification and quantification was also studied using triple fluorescent markers for H1-DNA, H2A and MPO (Supplementary Fig. 2A) demonstrating robust and similar trends in NETotic events in double-fluorescent populations of H1-DNA/H2A (Supplementary Fig. 2B) and H1-DNA/MPO (Supplementary Fig. 2C) after treatment with PMA with or without Ro 31-8220.

**Fig. 3:**
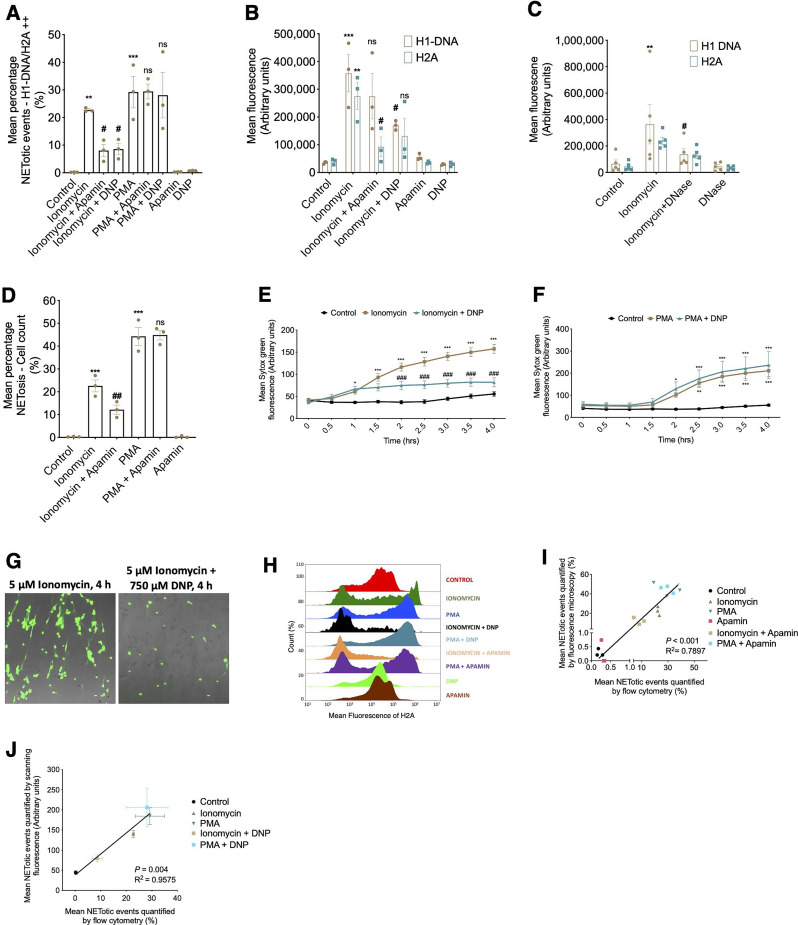
Flow cytometric detection of ionomycin-induced NETosis dependent upon SK channel activation and generation of mitochondrial ROS. Quantification of NETs by flow cytometry is accurate compared to established methods. (**A**) Mean percentages of double positive H1-DNA/H2A^++^ NETotic events analysed via flow cytometry after 3 h suspension culture without (control) or with 10 nM PMA and 5 µM ionomycin +/- 200 nM apamin and 750 µM (Ionomycin-specific NET inhibitors). (**B**) Mean fluorescence of H1- DNA/H2A analysed via flow cytometry after 3 h suspension culture without (control) or with 10 nM PMA and 5 µM ionomycin +/- 200 nM apamin and 750 µM (Ionomycin-specific NET inhibitors). (**C**) Mean fluorescence of H1-DNA and H2A with the addition of ionomycin and DNAse. *n* = 3 distinct cell donors. (**D**) Mean percentage NETosis after 4 h monolayer culture with the same treatments as used in (**A**), however NETosis was quantified from fluorescence microscopy counts. Data are expressed as means +/- SEM, *n* = 3 distinct cell donors. (**E** and **F**) Mean percentage NETosis after 4 h monolayer culture with the same treatments as used in (**A**), however NETosis was quantified using scanning fluorescence plate reading time course. *n* = 6 distinct cell donors. (**G**) Representative fluorescence microscopy images of neutrophils treated with ionomycin or ionomycin and DNP after 4 h of incubation, stained with Sytox Green. Scale = 10 μm. (**H**) Representative stagger offset histogram overlay of mean H2A fluorescence for each treatment. (**I**) Correlation between mean percentage of NETotic events quantified using fluorescence microscopy with events quantified by flow cytometry. Data are unpaired, *n* = 3 distinct cell donors. (**J**) Correlation between mean percentage of NETotic events quantified using scanning fluorescence with events quantified by flow cytometry. Data are unpaired, *n* = 6 distinct cell donors. Data are expressed as means +/- SEM. ** *P* < 0.01, *** *P* < 0.001 vs. corresponding control; # *P* < 0.05, ## *P* < 0.01 vs. corresponding Ionomycin; ns vs. PMA

Treatment with ionomycin increased the mean percentage double-positive H1-DNA/H2A population, which was reversed with pre-treatment of Apamin and DNP (Fig. 3A). As anticipated, the increase in NETotic events detected in PMA-treated cells was not reversed with the addition of these inhibitors. Similar to PMA-treated cells, ionomycin-treated cells exhibited an increase in mean fluorescence of H1-DNA and H2A but in contrast to PMA-pretreated cells, a reduction in H2A in cells pre-treated with apamin or DNP is observed. (Fig. 3B). H1-DNA decreased with the addition of DNAse (Fig. 3C). Reassuringly, quantification of NETs using fluorescent microscopy counting (Fig. 3D) and scanning fluorescence plate reading (Fig. 3E, F and G) demonstrated similar trends. The overlay of H2A histograms demonstrate a clear shift in H2A fluorescence intensity with the addition of ionomycin and ionomycin-specific inhibitors (Fig. 3H). Detection of NETosis using flow cytometry compared to fluorescence microscopy (Fig. 3I) and scanning fluorescence (Fig. 3J) showed a strong correlation, with R² values of 0.7897 (*P* < 0.001) and 0.9575 (*P* = 0.004), respectively, further supporting the reliability of our assay. By corroborating findings from current gold-standard techniques, including fluorescence microscopy and scanning fluorescence, across a broad range (< 0.5 to ~ 50%) of NETotic events, the flow cytometry-based assay demonstrates high sensitivity even when NETotic events are rare. Hence, the flow-cytometry based assay is at least an equivalent assay compared to existing gold-standard techniques of NET detection.

Collectively, these findings demonstrate that the flow cytometry assay can accurately identify PMA- and ionomycin-induced NETosis and its inhibition by their respective blockers, thereby confirming its ability to distinguish between different NETosis-inducing pathways. Furthermore, the assay is versatile and can be applied to unfixed cells as well as adapted for use with alternative NET markers.

### Flow-cytometry NET detection accurately detects *Staphylococcus aureus*-induced NETs, a biologically relevant NET-inducer

After validating the assay using non-physiological stimuli, we were interested in validating the assay using a biologically relevant stimulus. For this purpose, we chose *S. aureus* which has been demonstrated previously to induce NET formation.

**Fig. 4:**
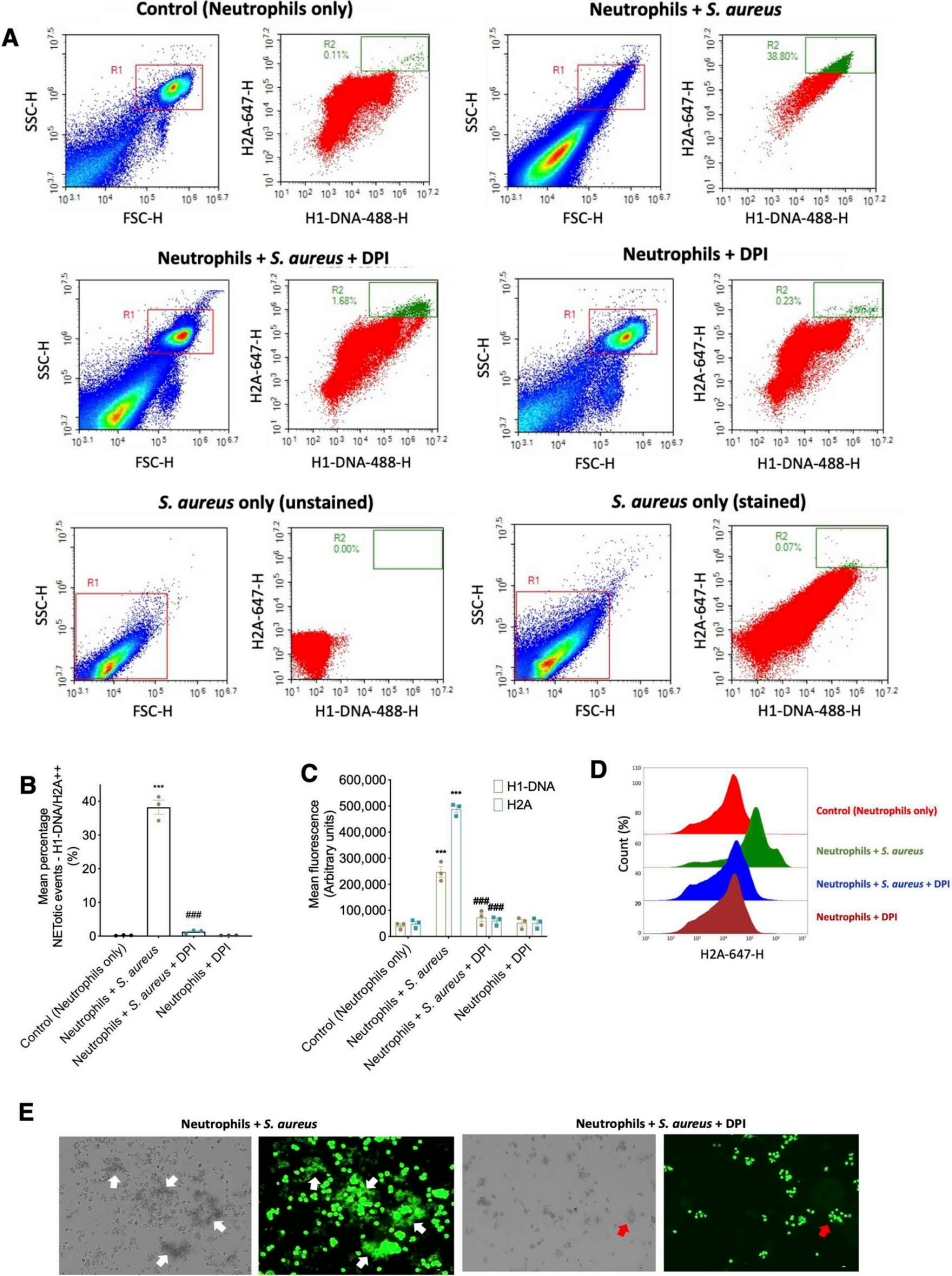
Flow cytometric detection and quantification of established *S. aureus*-induced NETosis dependent upon activation of NADPH oxidase. (**A**) Representative flow cytometry plots of NETotic cells identified using fluorescent markers for H1-DNA, H2A after exposure to *S. aureus* with or without DPI. (**B**) Mean percentages of double positive H1-DNA/H2A^++^ NETotic events analysed via flow cytometry after 3 h suspension culture of 500,000 neutrophils without (control) or with 10 million *S. aureus* bacteria (MOI 20) +/- 10 µM DPI. (**C**) Mean fluorescence of H1- DNA/H2A analysed via flow cytometry after 3 h suspension culture 500,000 neutrophils without (control) or with 10 million *S. aureus* bacteria (MOI 20) +/- 10 µM DPI. (**D**) Representative stagger offset histogram overlay of mean H2A fluorescence for each treatment. (**E**) Representative images of neutrophils incubated with S. aureus with or without DPI for 3 h stained with Sytox Green. White arrows indicate large clumps of bacteria caught within clouds of extracellular DNA. Red arrows indicate much smaller clumps of bacteria without clouds of extracellular DNA. Scale = 10 μm. Data are expressed as means +/- SEM, *n* = 3 distinct cell donors. ** *P* < 0.01, *** *P* < 0.001 vs. corresponding control; # *P* < 0.05, ## *P* < 0.01 vs. corresponding Ionomycin; ns vs. PMA

With exposure to *S. aureus*, the population of H1-DNA/H2A double-positive population significantly increased (Fig. 4A and B) and this was significantly reduced when pre-treated with DPI. This agrees with the literature that *S. aureus* induces NET formation via NADPH oxidase, which is inhibited by DPI [13, 52]. The mean fluorescence of both H1-DNA and H2A increased with the exposure to *S. aureus*, with a greater increase observed in H2A (Fig. 4C). This is in line with previous observation that H2A has greater cytotoxicity potential compared to mixture of histone proteins [53]. A clear shift in H2A fluorescence intensity is observed in an overlay of H2A histograms (Fig. 4D**)**. These data show that our flow-cytometry NET assay can detect NETs induced by *S. aureus*, a biologically relevant stimulus for NET formation.

### Flow-cytometry NET assessment effectively distinguishes neutrophils in activated state or exhibiting apoptotic or necrotic cell death

**Fig. 5:**
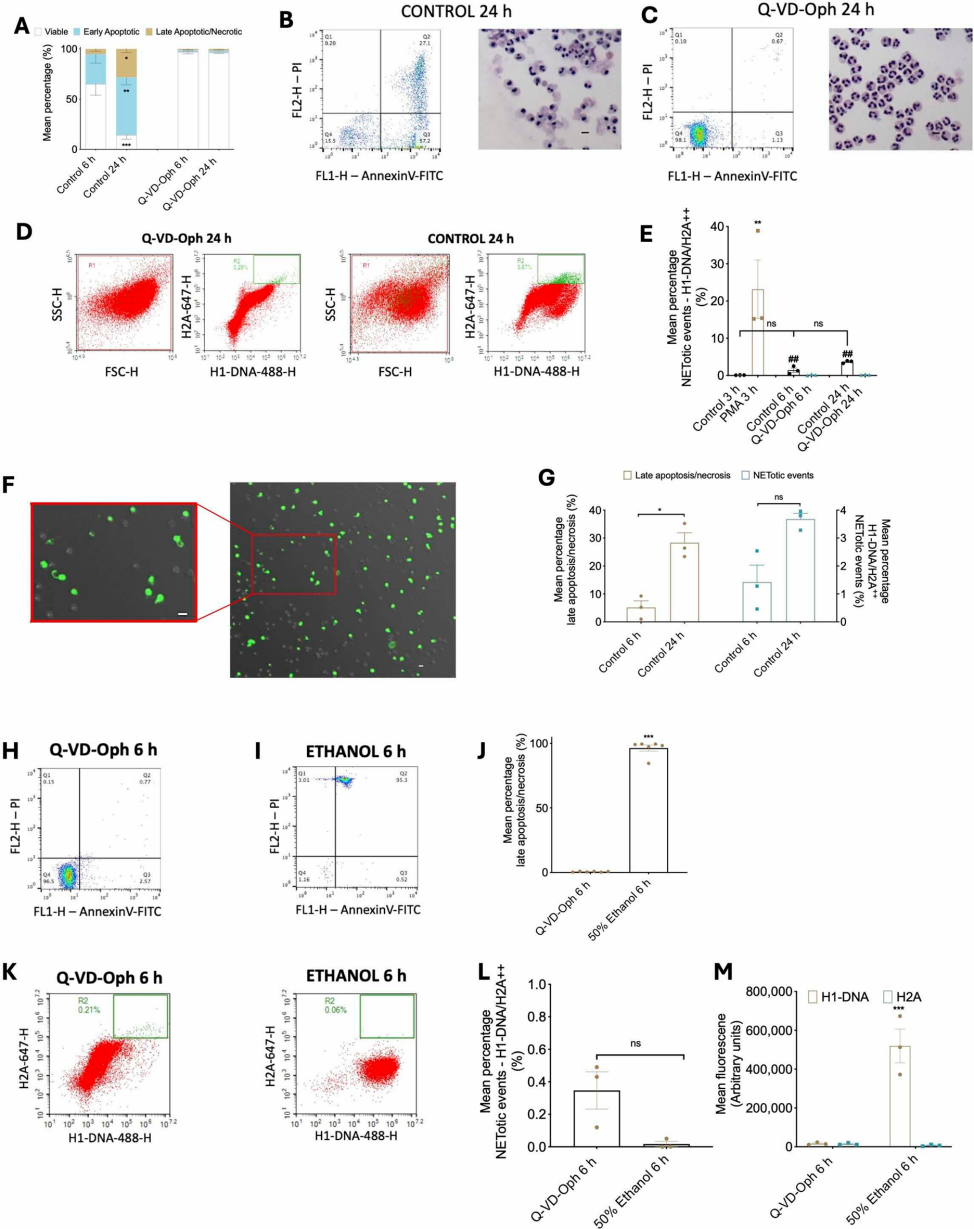
Flow cytometric detection of NETosis is distinctly separate to apoptotic and necrotic cell death. (**A**) Mean percentages of different proportion of viable (white), early apoptotic (green) and late apoptotic necrotic (red) cells after 6 and 24 h monolayer culture without (control) or with 10 µM Q-VD and stained with Annexin V-PI and analysed via flow cytometry. (**B**) Representative Annexin V-PI flow plots and cyto-centrifuge preparations of control 24 h. (**C**) Representative Annexin V-PI flow plots and cyto-centrifuge preparations of Q-VD 24 h. (**D**) Representative flow plots of Q-VD and control 24 h for H1-DNA and H2A. (**E**) Mean percentages of double positive H1-DNA/H2^++^ NETotic events analysed via flow cytometry after 3 h suspension culture without (control) or with 10 µM Q-VD. ** *P* < 0.01 vs. corresponding control; ## *P* < 0.01 vs. PMA; ns vs. control 3 h. (**F**) Representative images of control cells after 24 h culture demonstrating spontaneous NETs stained with Sytox green. Scale = 10 μm. (**G**) Mean percentages of late apoptotic-necrotic cells stained with AnnexinV- PI and analysed via flow cytometry (black) and mean percentages of double positive H1-DNA/H2A^++^ NETotic events analysed via flow cytometry (grey) after 6 culture without (control) or with 10 µM Q-VD. (**H**) Representative Annexin V-PI flow plots of Q-VD 6 h. (**I**) Representative Annexin V-PI flow plots of 50% ethanol 6 h. (**J**) Mean percentages of late apoptotic necrotic cells after 6 h monolayer culture with 10 µM Q-VD or 50% ethanol and stained with Annexin V-PI and analysed via flow cytometry. (**K**) Representative flow plots of Q-VD and ethanol 6 h for H1-DNA and H2A. (**L**) Mean percentages of double positive H1-DNA/H2A^++^ NETotic events analysed via flow cytometry after 6 h suspension culture with 10 µM Q-VD or 50% ethanol. (**M**) Mean fluorescence of H1- DNA/H2A analysed via flow cytometry after 6 h suspension culture with 10 µM Q-VD or 50% ethanol. Data are expressed as means +/- SEM, *n* = 3 distinct cell donors. * *P* < 0.05, ** *P* < 0.01, *** *P* < 0.001 unless stated above

One of the challenges of NET assessment is distinguishing NETs from other forms of cell death. Neutrophils undergo apoptosis within hours of in vitro culture, with the majority of cells undergoing apoptosis observed at 24 h (Fig. 5A and B). When treated with Q-VD, a pan-caspase inhibitor, the proportion of apoptotic cells was significantly diminished (Fig. 5A and C). On flow-cytometry, a small proportion of cells were observed to have double fluorescence H1-DNA/H2A at 6 and 24 h but this was significantly lower compared to cells treated with PMA (Fig. 5D and E). These cells were confirmed to demonstrate spontaneous NETosis using fluorescent imaging (Fig. 5F). Reassuringly, while the percentage of late apoptotic/necrotic cells increased from 6 to 24 h, flow-cytometric detection of NETotic events were not significantly increased, suggesting that flow-cytometry is sensitive at differentiating between late apoptotic/necrotic cells and NETotic cells (Fig. 5G). To further dissect the ability of our assay at distinguishing NETotic cells from apoptotic or necrotic cells, we incubated neutrophils stained with Annnexin V and PI for 6 h with Q-VD-Oph, an apoptosis inhibitor or 50% ethanol to induce necrosis. Cells pre-treated with Q-VD- demonstrated low PI and Annexin V indicative of living cells (Fig. 5H) while cells pre-treated with ethanol showed high PI and Annexin V indicative of late apoptosis or necrosis (Fig. 5I and J). Using our assay, both Q-VD-Oph and ethanol treated cells (Fig. 5K) had a low proportion of NETotic cells and there was no difference in the percentage of NETotic events between Q-VD and ethanol-pretreated cells (Fig. 5L). Notably, compared to PMA pre-treated cells (Fig. 2B) which had a significantly high mean fluorescence of H1-DNA and H2A, ethanol-treated cells exhibited high H1-DNA but not H2A (Fig. 5M). This demonstrates the importance of having dual targets of NET proteins as any assay dependent on detecting H1-DNA would have resulted in false positives. These data show that the flow-cytometry assay is effective at differentiating NETotic events from activated, apoptotic and necrotic states.

### Flow-cytometric detection and discrimination of activated and NET-otic cells using novel HNE-FQ probe

HNE-FQ is a novel peptide-based fluorogenic probe that has been recently reported to be sensitive and specific for detecting the presence of activated human neutrophil elastase [38]. Unlike fluorescently conjugated antibodies, which require cell permeabilization and cannot discriminate between active and inactive hNE isoforms, this probe can enter cells and selectively report enzymatic activation. It also provides advantages over traditional hNE detection methods such as immunostaining, ELISA, and Western blotting, as it enables real-time monitoring of hNE activity without the need for additional downstream processing. The specificity of HNE-FQ was confirmed by the lack of alteration in CD11b or CD62L expression (Supplementary Fig. 3A and B) or shape change that is typically observed with neutrophil activation (Supplementary Fig. 3C and D) when incubated with the probe for 3 h. On the contrary, when neutrophils were incubated with fMLF (Supplementary Fig. 3F), which was used as a positive control, HNE-FQ was effectively switched on, demonstrating specificity of the probe. Additionally, incubation of neutrophils with HNE-FQ for 3 h did not induce cell cytotoxicity, assessed using an LDH release assay (Supplementary Fig. 3F and G).

**Fig. 6:**
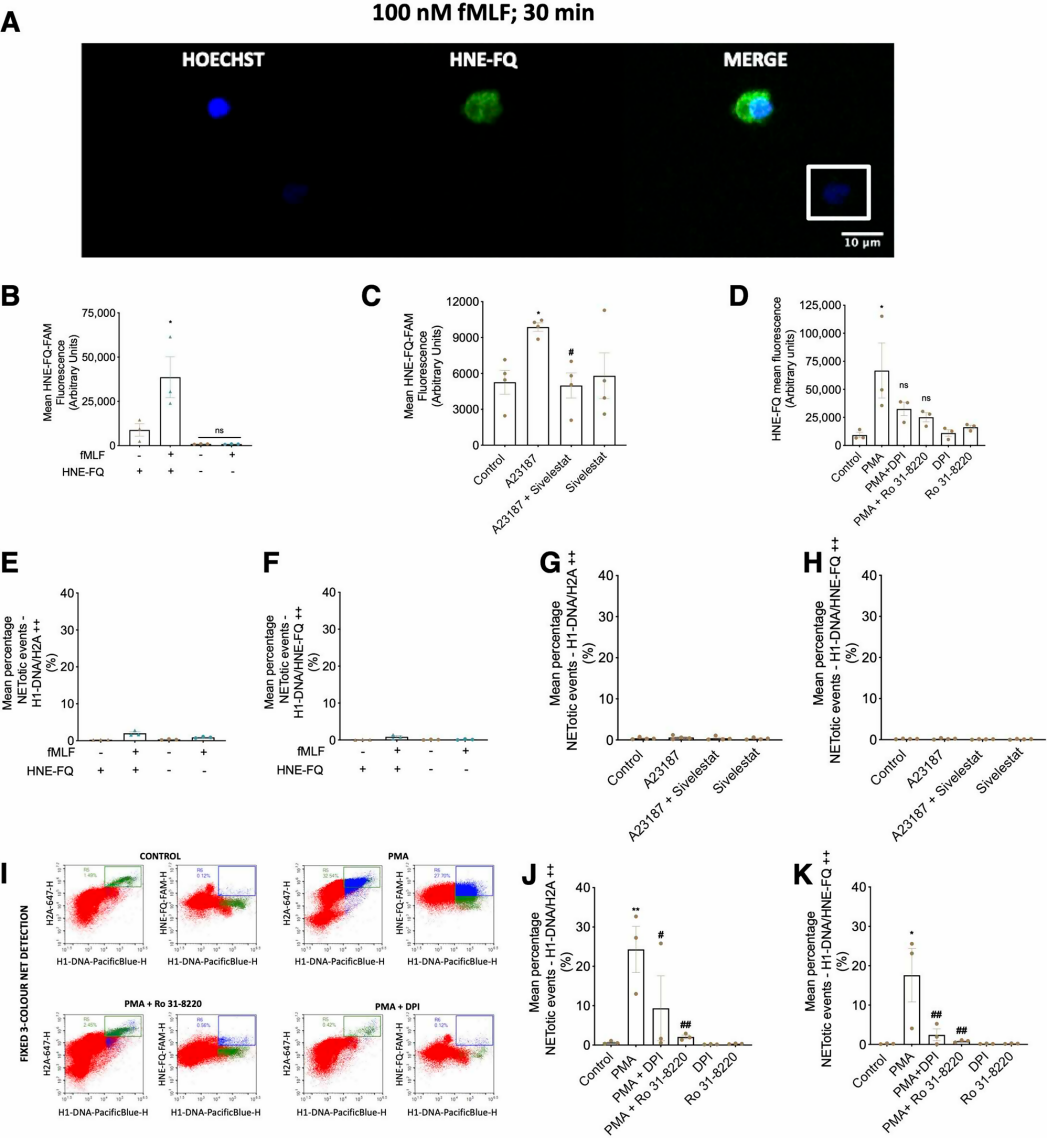
Flow cytometric detection and quantification of active human neutrophil elastase on established PMA-induced NETs using the novel fluorogenic peptide-based smartprobe HNE-FQ. (**A**) Representative image of neutrophil incubated with 100 nM fMLF for 30 min and stained with Hoechst and HNE-FQ. The activated neutrophil is fluorescent while the quiescent neutrophil (within the white box) is non-fluorescent. Scale = 10 μm. (**B**) Quantification of HNE-FQ mean fluorescence intensity with or without HNE-FQ and fMLF treatment for 30 min. *n* = 3 distinct cell donors. (**C**) Quantification of HNE-FQ mean fluorescence intensity with or without pre-treatment with 100 µM Sivelestat for 30 min and stimulated with fMLF for 30 min. *n* = 3 distinct cell donors. (**D**) Quantification of HNE-FQ mean fluorescence intensity with or without pre-treatment with 10 µM DPI or 1 µM Ro 31-8220 for 30 min and stimulated with PMA for 3 h. *n* = 3 distinct cell donors. (**E**, **F**) Percentages of highly double positive fluorescent cells for histone histone H1-DNA/histone H2A (**E**) and H1-DNA/HNE-FQ (**F**) of stained, unstained and fMLF-treated and untreated cells. *n* = 3 distinct cell donors. (**G**, **H**) Percentages of highly double positive fluorescent cells for histone histone H1-DNA/histone H2A (**G**) and H1-DNA/HNE-FQ (**H**) of neutrophils pre-treated with sivelestat (30 min) and 1 µM A23187-treated or untreated cells. *n* = 3 distinct cell donors. (**I**) Representative flow plots of MFIs of cells doubly positive of H1-DNA and H2A or HNE-FQ of untreated (control), neutrophil pre-treated with DPI and Ro (30 min) and stimulated with 10 nM PMA (3h). Cells of interest were gated for highly positive H1-DNA (> 10^4^ MFI) and at the edge of cell fluorescence for H2A or HNE-FQ. (**J**, **K**) Percentages of double positive NET populations for histone H1-DNA/H2A^++^ (**J**) and H1-DNA/HNE-FQ^++^ (**K**) in neutrophils pre-treated with DPI and Ro (30 min) and stimulated with PMA (3 h). *n* = 3 distinct cell donors. Values are expressed as mean MFI or percentages ± SEM. * *P* < 0.05, ** *P* < 0.01, *** *P* < 0.001

Mean fluorescence of HNE-FQ increased when cells were treated with fMLF, corroborating its ability to detect activated elastase (Fig. 6A and B). Further validation was carried out using other known neutrophil stimulators. Mean fluorescence increased when neutrophils were stimulated with A23187 and PMA, and this was reversed with the addition of the respective inhibitors, Sivelestat, DPI or Ro 31-8220 (Fig. 6C and D). We also confirmed that incubation of HNE-FQ with fMLF did not induce NETosis using our flow-cytometry assay when NETotic events were defined as cells double-positive for H1DNA/H2A and H1DNA/HNE-FQ (Fig. 6E and F). As expected, measurement of H1DNA/H2A and H1DNA/HNE-FQ double-positive populations after 30 min A23187 treatment did not reveal any changes suggesting that A23187 increased activated neutrophil elastase but did not induce NETosis (Fig. 6G and H).

Next, we investigated the utility of HNE-FQ as part of a three-colour panel for detecting NETotic events (Fig. 6I) and demonstrated that H1-DNA, H2A and HNE-FQ triple-positive cells demonstrated a significant increase with the addition of PMA which was reversed with DPI or Ro 31-8220 (Fig. 6J and K) confirming the utility of HNE-FQ as a marker for flow-cytometric detection of NETotic events.

## Discussion

We present a novel and reliable flow cytometry-based assay for detecting and quantifying NETs. The method is validated through multiple approaches and enables precise measurement of established NETotic pathways. Importantly, it is not influenced by neutrophil activation or by apoptotic and necrotic cell death - limitations that can affect currently accepted techniques.

Unlike several other reports which only included singlets as part of their flow cytometry gating strategy [32, 33, 35, 36], we opted for a simple and inclusive flow cytometric approach to detect/quantify NETotic events accounting for the ‘sticky’ properties of NETs and their morphological heterogeneity [54–56]. We chose a 3 h in vitro culture time for the majority of our experiments as it is well established that > 95% neutrophil viability is observed in control (untreated) samples after 4 h [57] ensuring minimal apoptotic or necrotic events. Three-hour incubations of neutrophils with either 10 nM PMA or 5 µM ionomycin were utilised as these conditions have been demonstrated to generate ample rates of NETosis [3, 46]. Using the same incubation period also allows comparability of our results with the existing literature.

By quantifying both H1-DNA and H2A double positive events, our assay is more accurate and less likely to detect false positives compared to the fluorescence cell counting or plate scanning fluorescence which only quantify NETs via use of a single DNA stain. Furthermore, our methodology is flexible in that additional NETosis markers can be detected to enable triple detection of key markers, including MPO and activated hNE via use of a novel fluorogenic peptide-based smartprobe (HNE-FQ), further increasing the specificity of NETs detected. Our methodology is designed to detect and quantify fixed NETotic material, allowing samples to be analysed by flow cytometry the following day. This approach allows a larger number of samples to be prepared and processed later, offering significant convenience to researchers.

Rapid, same-day detection and quantification of unfixed NETotic material can be achieved using Sytox Green in place of the monoclonal anti-DNA/Histone H1 antibody. This assay time is more comparable to traditional fluorescence microscopy counts. However, using this assay, we do lose a degree of NET-specificity during quantification as the nucleic acid stain Sytox Green replaces the anti-DNA/Histone H1 monoclonal antibody. This antibody is known to have affinity binding to decondensed chromatin released from NETs [42] and detects both extruded DNA and Histone H1 on NETs, hence providing increased NET-specificity. In terms of time requirements, our primary flow cytometry assay is comparable to immunocytochemistry, which is the qualitative gold standard for identifying hallmark NET-associated proteins (e.g., histones).

In tandem with H1-DNA detection, we also utilised a newly described fluorogenic peptide-based smartprobe, HNE-FQ, for the detection of activated hNE on NETs [38]. Using HNE-FQ, we were able to confirm the presence of activated hNE on externalized chromatin released from PMA-induced NETs and also quantify H1-DNA/hNE^++^ decorated NETotic events dependent upon activation of conventional PKC and NADPH oxidase. Detection of activated hNE on NETs provides crucial functional validation, confirming that the extruded DNA structures are enzymatically active and biologically relevant, rather than inert debris. While HNE-FQ exhibits green fluorescence, a second generation of probes based on HNE-FQ that emit in far-red has been developed, HNE-1F1Q, thereby providing greater flexibility for combining targets with different fluorescence profiles [58]. The field of NET detection/quantification via use of in-house fluorogenic probes will no doubt soon be in its ascendancy, moving the NET detection/quantification field away from more time-consuming antibody detection of key markers. Such probes have recently been described by Guerra and colleagues, where two FRET-based reporters were utilised to target and monitor DNA-bound hNE and cathepsin G on NETs found in sputum of cystic fibrosis patients [59]. Such probes could easily be included in our flow cytometry assay for the detection of NETs in unfixed cell samples.

Using the above methodologies, we validated the specificity of our assay at identifying NET formation by drugs that are well-understood to induce NET formation by two pathways, PMA by activation of PKC and ionomycin by the activation of SK channels. The reversal of NET formation with the addition of specific inhibitors of either pathway further solidify the validity of our assay. Besides using agents which are non-physiological, we also demonstrated the ability of our assay to detect NETs released as result of activation with *S. aureus*, a known biologically relevant simulator of NETs.

When comparing our principal flow cytometry assay for the quantification of fixed NETs to traditional NET quantification assays (e.g. fluorescence microscopy counts/algorithms and scanning fluorescence plate reading), length of assay time could be considered a limitation of our assay. This is in part due to centrifugation, fixation, blocking and antibody incubation steps post 3 h culture. Conversely, for fluorescence microscopy counts/algorithms NETs are ready to be analysed the same day, generally 15–30 min post 3 h culture via use of a vital DNA stain (e.g. Sytox Green). Similarly, scanning fluorescence plate reading gives NETotic readouts in real-time, whereby a vital DNA stain is co-cultured with neutrophils and mean fluorescence is measured at user defined intervals during the culturing period. However, as our assay utilises flow cytometry technology (takes away subjectivity inherent in NETotic counts/user designed algorithms) coupled with specific antibody detection of key NET markers (avoids false positives from other cell death processes), it is naturally superior, both qualitatively and quantitively, compared to these two methods. In addition, given the validation of our technique on unfixed cells, the processing time is comparable to traditional techniques.

In this proof-of-concept study, the assays were conducted on the Novocyte and ImageStreamX Mark II platforms, demonstrating consistency. However, it would have been ideal to validate assay consistency using other flow cytometers and with independent validation by external laboratories. We therefore acknowledge these as limitations of the current work and anticipate that wider adoption by the research community will provide additional validation of the assay’s generalisability and utility.

In future studies, this assay could be used to examine NET formation over time in response to stimulants or in clinical samples containing variable proportions of NETotic, apoptotic, and necrotic cells. Crucially, these populations can potentially be detected and imaged simultaneously using Annexin V (Pacific Blue), propidium iodide (red), histone H2A (far red), and H1–DNA (green) with imaging flow cytometry, thereby conserving valuable samples.

## Conclusions

In conclusion, we believe that our generated methodologies represent simple yet powerful tools for sensitive, reliable and efficient NETosis quantification and will serve to complement established NETosis methodologies. To the best of our knowledge, this is the most rigorous validation of a flow-cytometry based assay for NET detection and quantification.

## Supplementary Information

Below is the link to the electronic supplementary material.

**Supplementary Fig. 1** Flow cytometric detection and quantification of uplifted NETs post monolayer culture in unfixed cells. (**A**) Representative flow cytometry plots and images of NETotic cells post-monolayer culture after 3 h of incubation. Scale = 10 μm. (**B**) Quantification of mean percentages of double positive H1-DNA/H2A cells. (**C**) Representative flow cytometry plots of unfixed NETotic cells after 3 h of incubation. (**D**) Quantification of mean percentages of double positive Sytox green/H2A cells. Data are expressed as means +/- SEM, *n* = 3 distinct cell donors. ^**^P < 0.01 vs. control, ^##^*P* < 0.01 vs. PMA

**Supplementary Fig. 2** Fixed 3-colour flow cytometric detection and quantification of established PMA-induced NETosis. (**A**) Representative flow cytometry plots of NETotic cells identified using fluorescent markers for H1-DNA, H2A and MPO. (**B**) Quantification of mean percentages of double positive H1-DNA/H2A cells. (**C**) Quantification of mean percentages of double positive H1-DNA/MPO cells. Data are expressed as means +/- SEM, *n* = 3 distinct cell donors. ^**^P < 0.01 vs. control, ^##^*P* < 0.01 vs. PMA

**Supplementary Fig. 3** Confirmation of absence of neutrophil activation and minimal cytotoxicity using novel HNE-FQ. (**A**) Quantification of neutrophil CD62L expression with or without HNE-FQ and 100 nM fMLF treatment. *n* = 3 distinct cell donors. (**B**) Quantification of neutrophil CD11b expression with or without HNE-FQ and fMLF treatment. *n* = 3 distinct cell donors. (**C**) Shape change evaluation of neutrophils untreated or activated with fMLF in the presence or absence of HNE-FQ via quantification of standard deviation from the mean of the forward scatter using flow cytometry. *n* = 3 distinct cell donors. (**D**) Representative overlay of forward scatter histograms of untreated (red) or fMLF treated (green) cells. (**E**) Quantification of HNE-FQ MFI with or without the addition of fMLF. *n* = 3 distinct cell donors. (**F**) Quantification of the effect of a range of concentrations of HNE-FQ on cell toxicity after incubation for 3 h using a LDH release assay. 4% Triton-X was used as a positive control. *n* = 5 distinct cell donors. (**G**) Representative cytocentrifugation slide of neutrophils after incubation with 5 µM HNE-FQ for 3 h demonstrating healthy cells. Scale = 50 μm. Values are expressed as mean ± SEM. ^*^P < 0.05, ^**^P < 0.01, ^***^P < 0.001

## Data Availability

Data supporting findings and conclusions of this study is available within the article and Supplementary Information and from the corresponding author upon reasonable request.

## Abbreviations

COVID-19: Coronavirus disease 2019
DNA: Deoxyribonucleic acid
ELISA: Enzyme-linked immunosorbent assay
FBS: Fetal bovine serum
fMLF: N-Formylmethionine-leucyl-phenylalanine
FSC: Forward scatter
hNE: Human neutrophil elastase
LB: Luria-Bertani
LDH: Lactate dehydrogenase
MOI: Multiplicity of infection
MPO: Myeloperoxidase
NET: Neutrophil extracellular trap
PBS: Phosphate-buffered saline
PMA: Phorbol 12-myristate 13-acetate
PRP: Platelet-rich plasma
RPMI: Roswell Park Memorial Institute
SLE: Systemic Lupus Erythematosus
SSC: Side Scatter
