# Supplementary figures and images for: Simplified flow cytometric quantification of human neutrophil extracellular traps (NETs)
Source: J Inflamm (Lond). 2026 Feb 18;23:6. doi: 10.1186/s12950-026-00490-0 (PMC12947343; doi:10.1186/s12950-026-00490-0)

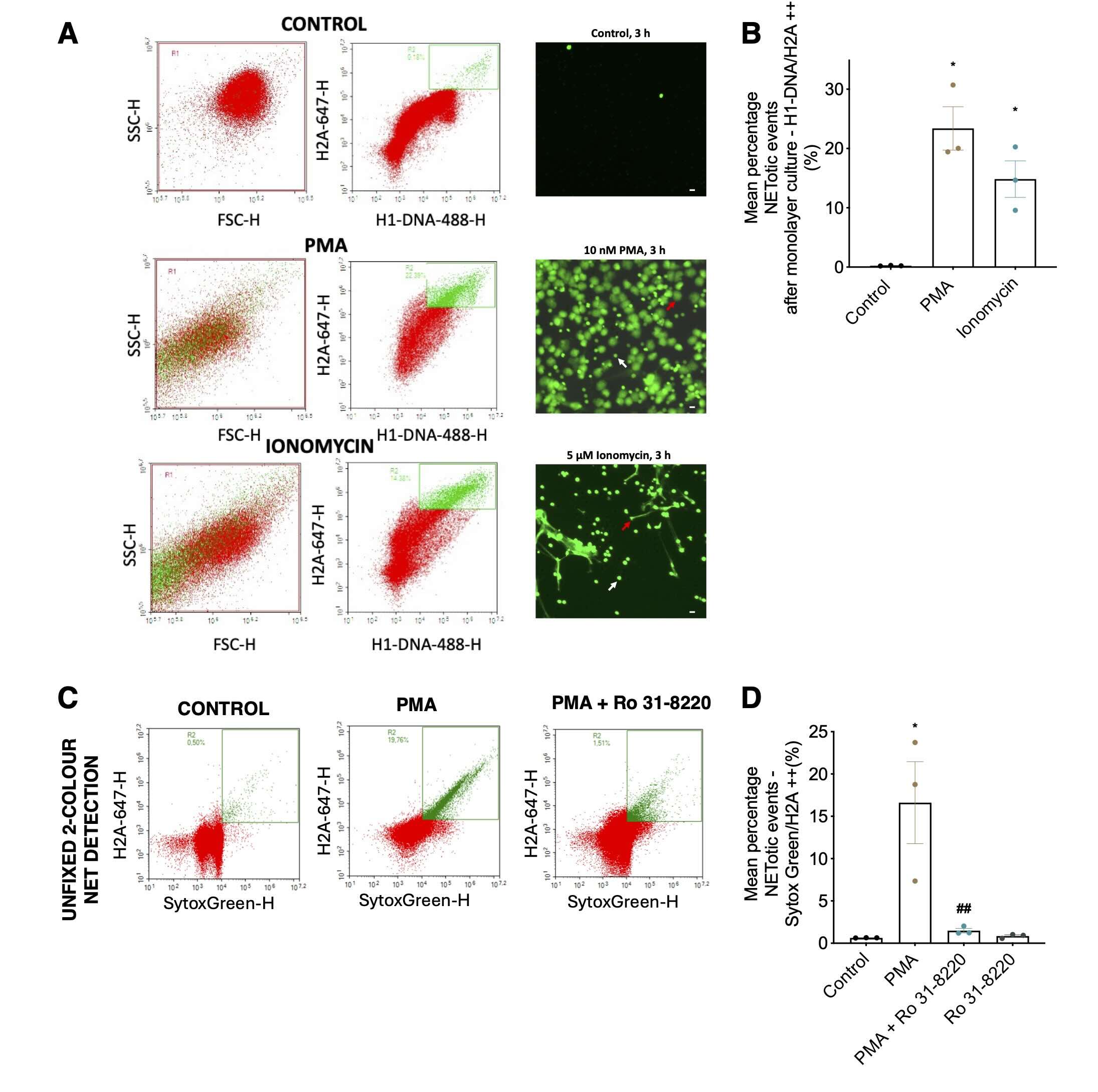

Supplement: Supplementary file 1 — Supplementary Fig. 1 Flow cytometric detection and quantification of uplifted NETs post monolayer culture in unfixed cells. (A) Representative flow cytometry plots and images of NETotic cells post-monolayer culture after 3 h of incubation. Scale = 10 μm. (B) Quantification of mean percentages of double positive H1-DNA/H2A cells. (C) Representative flow cytometry plots of unfixed NETotic cells after 3 h of incubation. (D) Quantification of mean percentages of double positive Sytox green/H2A cells. Data are expressed as means +/- SEM, n = 3 distinct cell donors. **P < 0.01 vs. control, ##P < 0.01 vs. PMA [file 12950_2026_490_MOESM1_ESM.jpeg]

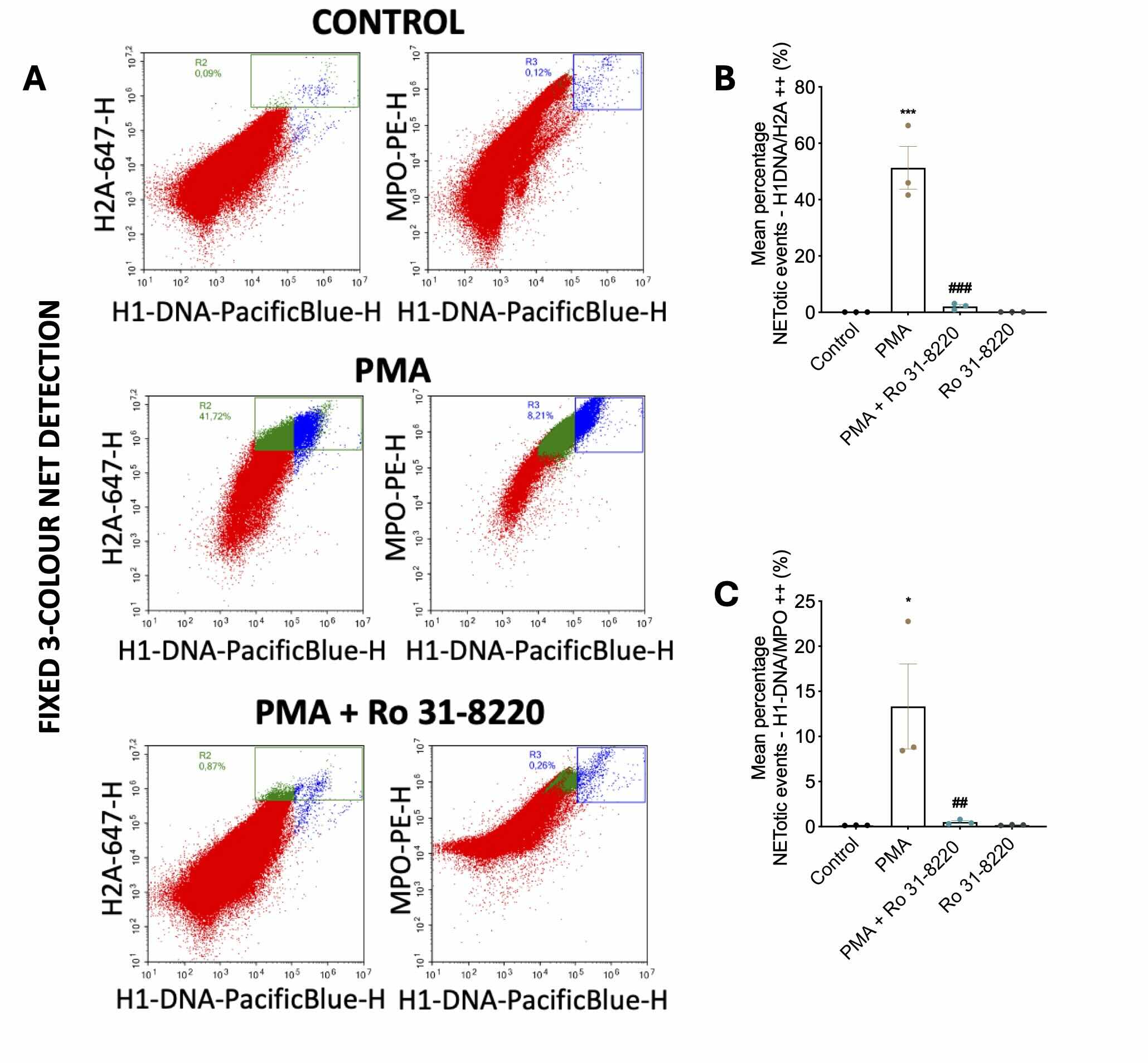

Supplement: Supplementary file 2 — Supplementary Fig. 2 Fixed 3-colour flow cytometric detection and quantification of established PMA-induced NETosis. (A) Representative flow cytometry plots of NETotic cells identified using fluorescent markers for H1-DNA, H2A and MPO. (B) Quantification of mean percentages of double positive H1-DNA/H2A cells. (C) Quantification of mean percentages of double positive H1-DNA/MPO cells. Data are expressed as means +/- SEM, n = 3 distinct cell donors. **P < 0.01 vs. control, ##P < 0.01 vs. PMA [file 12950_2026_490_MOESM2_ESM.jpeg]

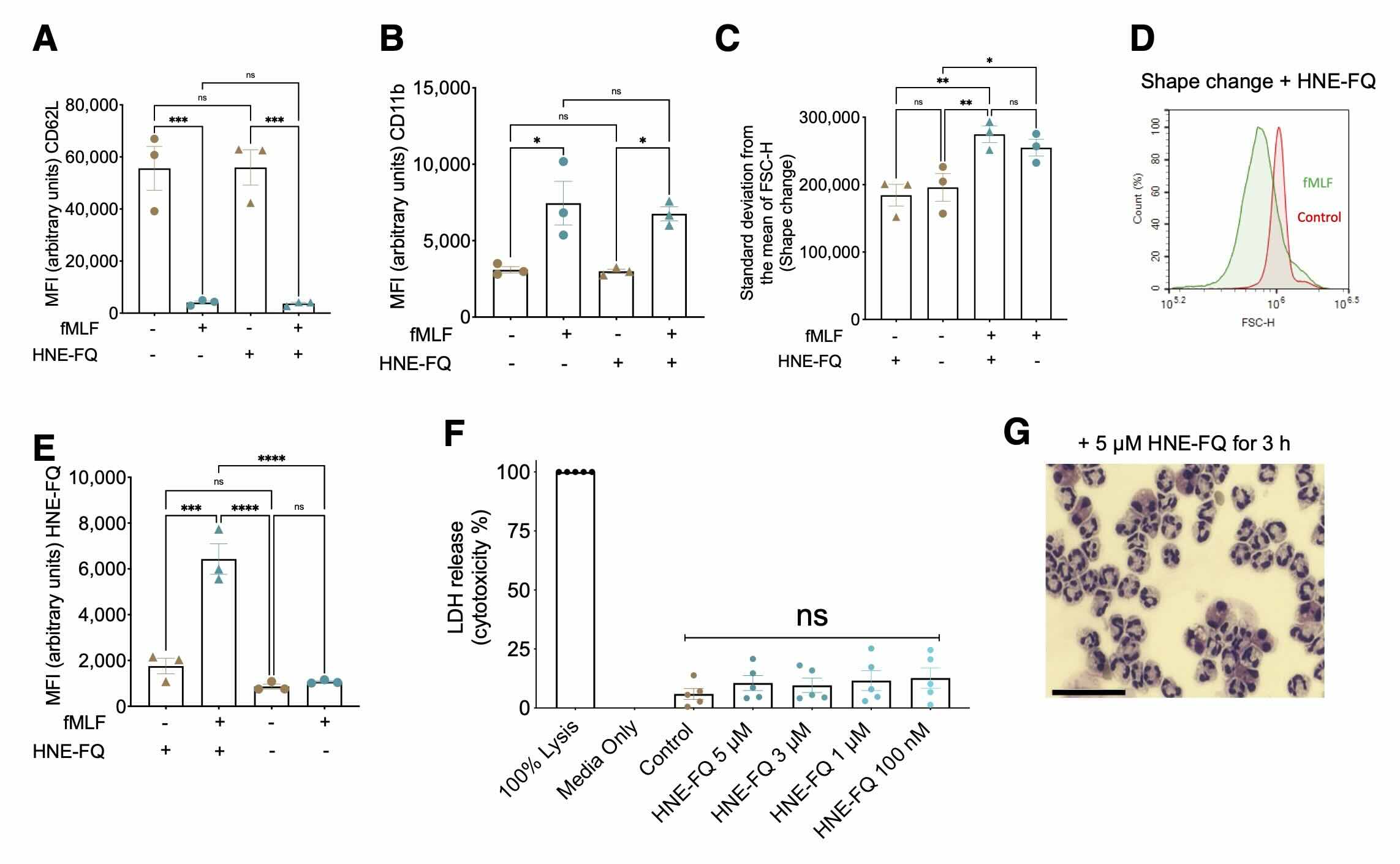

Supplement: Supplementary file 3 — Supplementary Fig. 3 Confirmation of absence of neutrophil activation and minimal cytotoxicity using novel HNE-FQ. (A) Quantification of neutrophil CD62L expression with or without HNE-FQ and 100 nM fMLF treatment. n = 3 distinct cell donors. (B) Quantification of neutrophil CD11b expression with or without HNE-FQ and fMLF treatment. n = 3 distinct cell donors. (C) Shape change evaluation of neutrophils untreated or activated with fMLF in the presence or absence of HNE-FQ via quantification of standard deviation from the mean of the forward scatter using flow cytometry. n = 3 distinct cell donors. (D) Representative overlay of forward scatter histograms of untreated (red) or fMLF treated (green) cells. (E) Quantification of HNE-FQ MFI with or without the addition of fMLF. n = 3 distinct cell donors. (F) Quantification of the effect of a range of concentrations of HNE-FQ on cell toxicity after incubation for 3 h using a LDH release assay. 4% Triton-X was used as a positive control. n = 5 distinct cell donors. (G) Representative cytocentrifugation slide of neutrophils after incubation with 5 µM HNE-FQ for 3 h demonstrating healthy cells. Scale = 50 μm. Values are expressed as mean ± SEM. *P < 0.05, **P < 0.01, ***P < 0.001 [file 12950_2026_490_MOESM3_ESM.jpeg]
